# Chronic liver disease and hepatic calcium-oxalate deposition in patients with primary hyperoxaluria type I

**DOI:** 10.1038/s41598-022-19584-9

**Published:** 2022-10-06

**Authors:** Pia Recker, Bodo Bernhard Beck, Przemyslaw Sikora, Heike Göbel, Markus Josef Kemper, Angel Nazco, Cristina Martin-Higueras, Bernd Hoppe

**Affiliations:** 1 Department of Pediatric and Adolescent Medicine, Division of Pediatric Nephrology, University Hospital Cologne, Cologne, Germany; 2 Department of Pediatric and Adolescent Medicine, HELIOS University Hospital Wuppertal, Wuppertal, Germany; 3 Institute of Human Genetics, University Hospital Cologne, Cologne, Germany; 4 Department of Pediatric Nephrology, Lublin Medical University, Lublin, Poland; 5 Department of Pathology, University Hospital Cologne, Cologne, Germany; 6 Department of Pediatric and Adolescent Medicine, Asklepios Klinik Hamburg, Hamburg, Germany; 7 Department of Pathology, Nuestra Señora de Candelaria University Hospital, Tenerife, Spain; 8 Department of Basic Medical Sciences, Centre for Biomedical Research in Rare Diseases (CIBERER), Institute of Biomedical Technologies (ITB), University of La Laguna, Tenerife, Spain; 9 German Hyperoxaluria Center, c/o Kindernierenzentrum Bonn, Bonn, Germany

**Keywords:** Nephrology, Kidney diseases, Renal calculi, Liver fibrosis

## Abstract

Patients with primary hyperoxaluria type I (PH I) are prone to develop early kidney failure. Systemic deposition of calcium-oxalate (CaOx) crystals starts, when renal function declines and plasma oxalate increases. All tissue, but especially bone, heart and eyes are affected. However, liver involvement, as CaOx deposition or chronic hepatitis/fibrosis has never been reported. We examined liver specimen from 19 PH I patients (aged 1.5 to 52 years at sample collection), obtained by diagnostic biopsy (1), at autopsy (1), or transplantation (17). With polarization microscopy, birefringent CaOx crystals located in small arteries, but not within hepatocytes were found in 3/19 patients. Cirrhosis was seen in one, fibrosis in 10/19 patients, with porto-portal and nodular fibrosis (n = 1), with limitation to the portal field in 8 and/or to central areas in 5 patients. Unspecific hepatitis features were observed in 7 patients. Fiber proliferations were detectable in 10 cases and in one sample transformed Ito-cells (myofibroblasts) were found. Iron deposition, but also megakaryocytes as sign of extramedullary erythropoiesis were found in 9, or 3 patients, respectively. Overall, liver involvement in patients with PH I was more pronounced, as previously described. However, CaOx deposition was negligible in liver, although the oxalate concentration there must be highest.

## Introduction

Currently three types of primary hyperoxaluria (PH I—III) can be accurately defined by substrate analysis in the urine and then confirmed by genetic testing^1–3^. They constitute rare autosomal-recessive inborn errors of hepatic glyoxylate metabolism resulting in extremely increased endogenous oxalate synthesis. Since oxalate cannot be metabolized in mammals it is primarily eliminated via the kidneys. The high urinary oxalate excretion (typically > 0.8–1 mmol/1.73m^2^/24 h, normal < 0.5), results in calcium-oxalate (CaOx)-deposition within the renal parenchyma (nephrocalcinosis/oxalosis) and/or recurrent stone formation (urolithiasis), the clinical hallmarks of the primary hyperoxalurias^1–5^.

Type I primary hyperoxaluria (PH I, MIM 259,900; 604,285), is caused by deficient or absent activity of liver-specific alanine:glyoxylate aminotransferase (AGT^6^). It represents the most frequent and most severe type of PH. In a substantial subgroup (about 10%) of patients, end-stage kidney failure (ESKF) occurs early, e.g. in the first year of life (infantile oxalosis). In the long-run, the vast majority of adults will develop ESKF between the third and fifth decade of life^7,8^. With advanced renal failure the disease turns into a lethal multi-systemic disorder making renal replacement therapy and, so far, subsequent liver-kidney transplantation necessary. Recently, however, a new treatment option, at least for PH I was reported. An RNA interference medication (Oxlumo™, Alnylam Pharmaceuticals, USA) blocks a specific enzyme related to the oxalate production (glycolate oxidase) as substrate reduction therapy. It is available for repeated subcutaneous injection and may prevent the necessity for liver transplantation^9^. Next to that current standard of care treatment with vitamin B6 (VB6) reduces oxalate production in a subgroup of patients and then can even lead to normalization of urinary oxalate excretion^2^. Therefore, also this group of patients would not need liver, but only kidney transplantation, if necessary.

Primary hyperoxaluria type II (PHII, MIM 260,000, 604,296) occurs as a result of deficient (ubiquitous) glyoxylate reductase/hydroxypyruvate reductase (GRHPR) enzyme activity^10^. In general type II shows a milder clinical course, however, recent data showed, that up until 50% of PH II patients are in chronic kidney failure (CKD) and 25% experience ESKF^11^. PH II accounts for less than 10% of all PH cases in the PH registries. In case of ESKF isolated kidney transplantation is mainly regarded as sufficient but there are emerging reports, that also liver-kidney transplantations are necessary in problematic cases^12,13^.

Primary hyperoxaluria type III (PH III, MIM 613,616, 613,597) is known since 2010 when a third causative gene (*HOGA1*) coding for hydroxy-oxo-glutarate aldolase type 1 was first described^14,15^. Patients with PH III present with frequently recurring kidney stones even in early childhood, remain stone formers also in adulthood and, in the contrary to previous reports, can eventually develop CKD, but also ESKF^16,17^. However, no transplant procedure has been reported so far in PH III.

**Figure 1:**
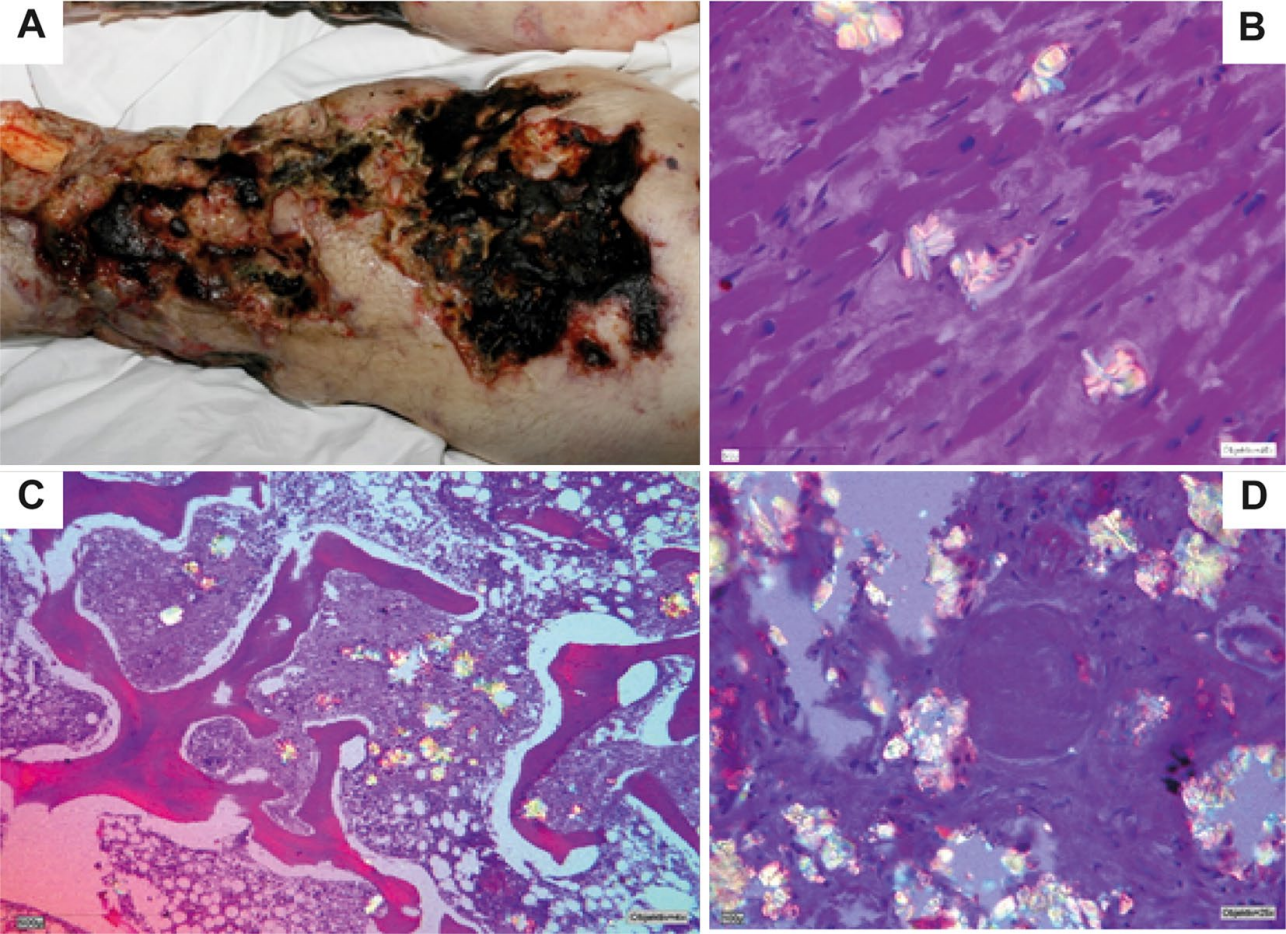
Severe systemic oxalosis in patient 11 (autopsy). (**A**) The oxalate crystals in the skin caused vascular ischemic necrosis, which became superinfected in the course of the disease. (**B**) The damage of the myocardium, caused by CaOx deposition, finally led to heart failure and consecutively death. (**C**/**D**) Birefringent CaOx deposits in the bone marrow and in the kidney. CaOx = calcium-oxalate.

In the face of severely compromised renal function in PH, and here especially in PH I, plasma oxalate (Pox) concentration rises and exceeds the supersaturation threshold at levels > 30 µmol/L (normal: < 7.4 µmol/l^18^) what will invariably result in systemic deposition of calcium-oxalate salts (oxalosis). The devastating effects of oxalosis are causing cardiomyopathy, cardiac conduction disturbances, heart block, vasculopathy, treatment resistant anemia, debilitating oxalate osteopathy and retinopathy (Fig. 1). If untreated early death has also been well documented^1,2^. Thus when advanced, the primary hepatic defect which first manifests within the urogenital tract becomes a devastating multi-systemic disorder.

Interestingly, although the liver carries the cause and its transplantation is the only curative treatment option in PH I, there has been no systematic evaluation of liver tissue to document potential other liver affection in PH I, than the enzyme defect. It is well known that prolonged time on hemodialysis (HD) negatively affects outcome and that progressive general vasculopathy in systemic oxalosis is one of the main factors contributing to the moribund condition of advanced disease^19^.

Also, patients with severe secondary hyperoxaluria, e.g. patients with Crohn’s disease, but also patients with long-term dialysis experience elevated Pox levels and hence are on risk to develop systemic oxalate deposits^20^. However, the cause of hyperoxaluria is not an enzymatic defect in the liver.

We report the first systematic analysis of liver tissue findings in PH I patients suffering from ESKF and systemic oxalosis across all age groups from infantile oxalosis to late onset disease.

## Material and methods

Liver tissue samples were taken from 19 patients with PH I, aged 1.5–52 years at time of specimen collection, and their status of systemic oxalosis was described. Genetic background was known in all patients (Table 1). Clinical information on age at first symptom, age at diagnosis, age at ESKF, mode and time on dialysis and transplantation procedures as well as current clinical status are shown in Table 1. The study was approved by the local institutional review boards and patients and/or their care takers had signed an informed consent (IRB approvals Cologne 06-231; Bonn 113/14, both Germany, Lublin, Poland KE-0254/118/2016 and Tenerife, Spain CHUC_2020_79). Clinical data were stored in a secured and encrypted online database (www.ph-registry.net). All study related methods were carried out in accordance with relevant guidelines and regulations (Declaration of Helsinki).

**Table 1:** Clinical data of all patients examined.

Pat. No	Fam. No	Sex	*AGXT* Genotype HGVS nucleotide (c) protein (p)	Type of *AGXT* mutation	1st Symptom (years)	Diagnosis (years)	ESKF (years)	Dialysis time (months) and mode	VB6 prior ESKF	Age at LTx	Pox before and promptly after study related Tx (µmol/l)	Transplantation type and year	Outcome
1	F1	m	c.409C > T; c.508G > A p.Gln137* p.Gly170Arg #	Nonsense Missense	0.1	0.2	0.2	15 PD/HD	No	11 mo	174.7 / 24.26	LTx related 2007	Death at 2.3 years, septicemia
2	F2	f	c.1079G > A; c.1079G > A p.Arg360Gln p.Arg360Gln	Missense Missense	0.1	0.4	0.2	17 PD/HD	No	1 y 5 mo	136.4 / 15.81	LKTx and re LKTx in 06/2011 3.LKTx in 11/2018	SOF, eGFR 95 ml/min
3	F3	m	c.331C > T; c.454 T > A p.Arg111* p.Phe152Ile #	Nonsense Missense	0.5	0.5	0.5	12 PD/HD	No	1 y 6 mo	143.9 / 33.43	LKTx related 08/2008, 2nd KTx in 2021	HD from 01/20–03/21; now SOF, eGFR 77 ml/min
4	F4	m	c.508G > A; c.33dupC p.Gly170Arg # p.(Lys12Glnfs*156)	Missense Frameshift	0.3	0.3	0.3	15 PD/HD	No	1 y 6 mo	116.25 / 23.33	LKTx 2007	SOF, eGFR 26.3 ml/min
5	F5	m	c.533G > A; c,533G > A p.Cys178Tyr p.Cys178Tyr	Missense Missense	2.5	5.8	2.5	62 PD/HD	No	5 y 2 mo	n.a./n.a	iKTx 05/2001, LKTx 09/2003	SOF, eGFR 60 ml/min at 27 years
6	F6	m	deletion of exons 6–8 deletion of exons 6–8 p.Ile200Alafs*29 p.Ile200Alafs*29	Large deletion Large deletion	1.1	8.3	6.8	28 HD	No	9 y 2 mo	n.a./15.78	iKTx 04/2008, LKTx 05/2009	KTx lost at age 18 years, since on HD, stable LTx function at 23 years
7	F7	f	c.508G > A: c.508G > A p.Gly170Arg # pGly170Arg #	Missense Missense	2	4.7	5.4	72 PD/HD	No	11 y 4 mo	114.7 /n.a	Attempted LKTx 05/2009	Death at 11.4 years
8	F6	f	deletion of exons 6–8 deletion of exons 6–8 p.Ile200Alafs*29 p.Ile200Alafs*29	Large deletion Large deletion	11.6	13.5	14.5	6 HD	Yes	14 y 11 mo	n.a./n.a	LKTx 07/2008	SOF, eGFR 92 ml/min at 29 years
9	F8	m	c.508G > A; c.508G > A p.Gly170Arg # pGly170Arg #	Missense Missense	2	17	14	156 HD	No	17 y	n.a./n.a	iKTx 2000§, LKTx 2003§, iKTx 2005§	KTx lost at 22 years, sudden death after 6 months on HD due to cardiac arrest, with stable LTx function
10	F9	f	c.33delC; c,33delC p.Lys12Argfs*34 p.Lys12Argfs*34	Frameshift Frameshift	5	13	10	108 HD	No	19 y	n.a./n.a	iKTx 1996, LKTx 2002	Sudden death at 33 years due to cardiac arrest with SOF, eGFR 47 ml/min
11	F10	f	c.508G > A c.508G > A p.Gly170Arg # pGly170Arg #	Missense Missense	0.3	28	28	42 HD	No	31 y 17 mo	80.8 / n.a	None	Death 31.6 years
12	F11	f	c.508G > A c.508G > A p.Gly170Arg # pGly170Arg #	Missense Missense	6	43	40.5	20 HD	No	42 y	27.98 / 8.74	LKTx 2006	Death in 2011 due to septicemia
13	F12	f	c.508G > A; c.1084G > A p.Gly170Arg # p.Gly362Ser	Missense Missense	42	48,1	47.9	15 HD	No	50 y 2 mo	112.9 / 5.16	LKTx 1/2009	SOF, eGFR 40 ml/min
14	F13	m	c.449 T > C; c.1110_1111delCG p.Leu150Pro p.(Asn372Cys*40)	Missense Frameshift	0.1	36	47	17 HD	No	48 y 11 mo	130.34 / 67.13	LTx in 2017	Death at 49 years, septicemia
15	F14	m	c.733_734del AA; c.733_734del AA p. (Lys245Valfs*9) p. (Lys245Valfs*9)	Frameshift Frameshift	0.7	12	20	51 HD	No	23 y 1 mo	74.47 / 22.59	LTX in 2018 and KTx in 2019	Back on HD in 2020
16	F15	f	c.122G > A; c.508G > A; p.Gly41Glu p.Gly170Arg #	Missense Missense	0.4	13	19	15 HD	Yes	20 y	92.46 / 9.11	LKTx in 2012	SOF, eGFR 74 ml/min
17	F16	f	c.508G > A; c.846-3C > G p.Gly170Arg # p.?	Misssense Splicing	2	15	22	72 HD	Yes	30 y 3 mo	110.93 / 84.03 after LTx and 42.78 after KTx	KTx in 2013 and failure in 2014, LTx in 2019 and KTx in 2020	SOF, eGFR 58.8 ml/min
18	F17	f	c.560C > T; c.560C > T p.Ser187Phe p.Ser187Phe	Missense missense	0.1	0.5	16	46 HD	Yes	19 y 6 mo	82.48 / n.a	LTx in 2019 and KTx in 2020	SOF, eGFR 37.4 ml/min
19	F18	m	c.731 T > C; c.731 T > C p.Ile244Thr # p.Ile244Thr #	Missense missense	6.2	6.8	22.5	24 HD	Yes	25 y	n.a / 11.0 after LTx and 17.4 after KTx	LTx in 2020 with 2^nd^ liver one day later after ALF, KTx in 06/2021	SOF, eGFR 46 ml/min

iKTx = isolated kidney transplantation, LTx = liver transplantation, LKTx = liver/kidney transplantation; all transplantation cadaveric unless otherwise stated; PD = peritoneal dialysis, HD = hemodialysis, ALF = acute liver failure, eGFR = estimated glomerular filtration rate (CKD-EPI formula), SOF = stable organ function, Pat. = patient, Fam. = family, VB6 = vitamin B6, hz = homozygous, y = year, mo = months, f = female, m = male.

^§^Immediate kidney graft failure and need for ongoing hemodialysis treatment.

^#^Indicates potential pyridoxine sensitive *AGXT* missense mutations.

Most tissue (n = 17) was available after combined or sequential liver-kidney transplantation. In one case each, liver tissue was taken at diagnostic biopsy or at autopsy, the latter providing samples of other organs in addition to the liver (Fig. 1). With the exception of the biopsy specimen, we had received multiple large area cuts of approximately 5 cm in diameter from the explanted liver. All pieces available were later histologically examined.

The tissue blocks were fixed in formaldehyde, dehydrated, embedded in paraffin, sectioned at 1–2 µm for routine staining and the following five routine stains were applied: Hematoxylin–eosin (H&E) staining, Van Gieson's, Gomorri, Periodic acid-Schiff (PAS) and Iron stain.

Additionally, some tissue was investigated further using histochemical stains (chlor-acetate-sterase) and immuno-histochemistry methods, e.g. extramedullary hematopoiesis with CD61 (patient 5 and 7), muscle and myofibroblasts with α-actin (patient 3) and bile duct proliferation with CK7 (patient 3).

To look for CaOx depositions within the liver, the hematoxylin–eosin staining of all specimens were examined with a polarization filter for the typical birefringent crystals.

## Results

All clinical and follow up data of the patients are depicted in Table 1. It is to mention that diagnosis was delayed in 13/19 patients, with a minimum of 1.9 to a maximum of 37 years between first symptom and diagnosis. Patient 11 was only diagnosed with severe systemic oxalosis and in a disastrous condition, although her sister had already died due to PH I after a combined liver-kidney transplantation (LKTx) years before. No family screening had been performed and diagnosis was only established when she had developed advanced systemic oxalosis (Fig. 1).

Seven (two children, five adults) of the 19 patients died, two promptly after transplantation (Tx) procedures, two post septicemia 1 and 5 years after transplantation, two patients without obvious transplant-related reason in long-term follow up and one patient (patient 11) meanwhile on maintenance HD, but not transplantable because of severe systemic oxalosis. Combined LKTx as first Tx procedure was performed in 7 patients and it was attempted in one young patient (patient 7), but she died at Tx procedure. Sequential LKTx was performed in 4 patients. One patient lost kidney function due to recurrent CaOx deposition promptly (10 days) after the kidney transplantation (KTx). Three patients needed a second liver grafting, one of them twice (Table 1).

Only LTx was performed in two patients, who later died after transplantation (patient 1 and 14). In 4 patients KTx was performed before LTx or LKTx, as the correct diagnosis was not available at time of first transplantation. One further VB6 sensitive patient (17) had received an isolated KTx as first Tx procedure. The transplant was lost because of mal-compliance to medications. Six years later she received a sequential liver and then kidney transplantation.

**Figure 2:**
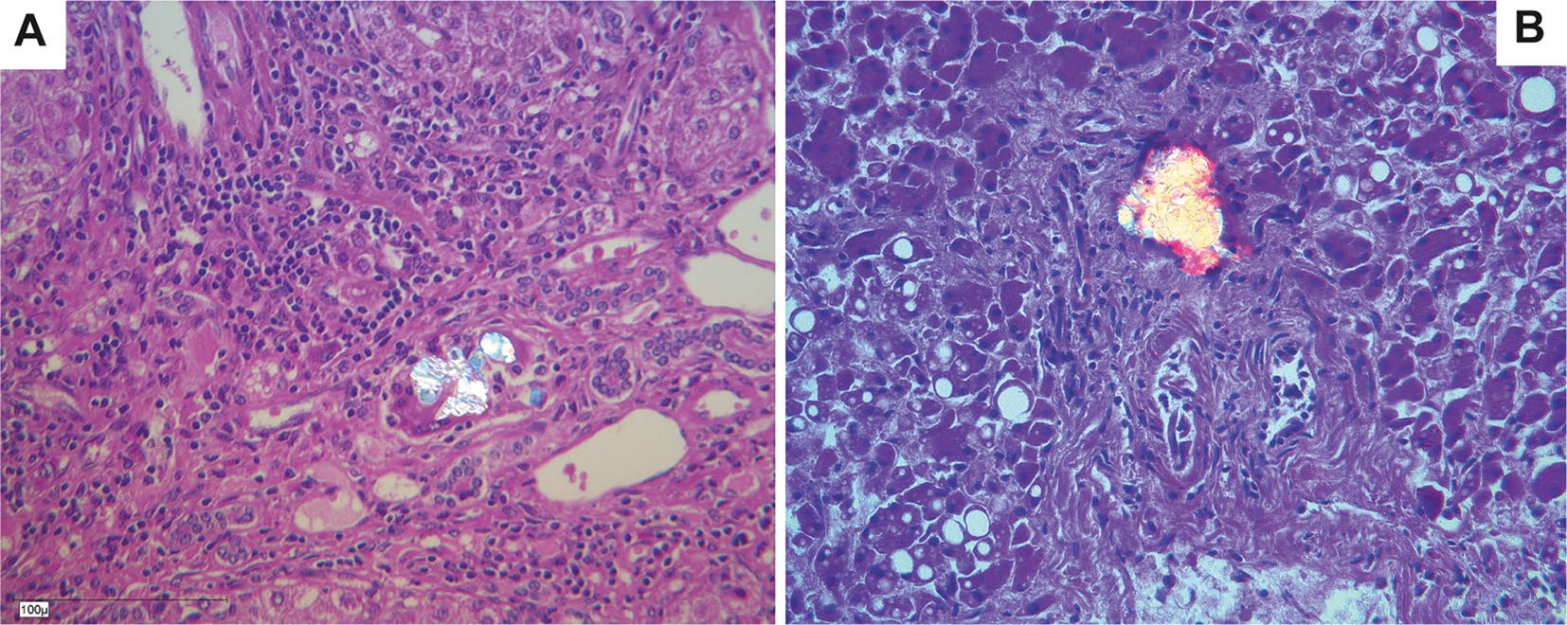
Calcium-oxalate deposition in the liver as typical birefringent crystals detected by polarization microscopy in two patients (**A**) patient 10, (**B**) patient 11. The crystals were located in small arteries, but not within hepatocytes.

**Figure 3:**
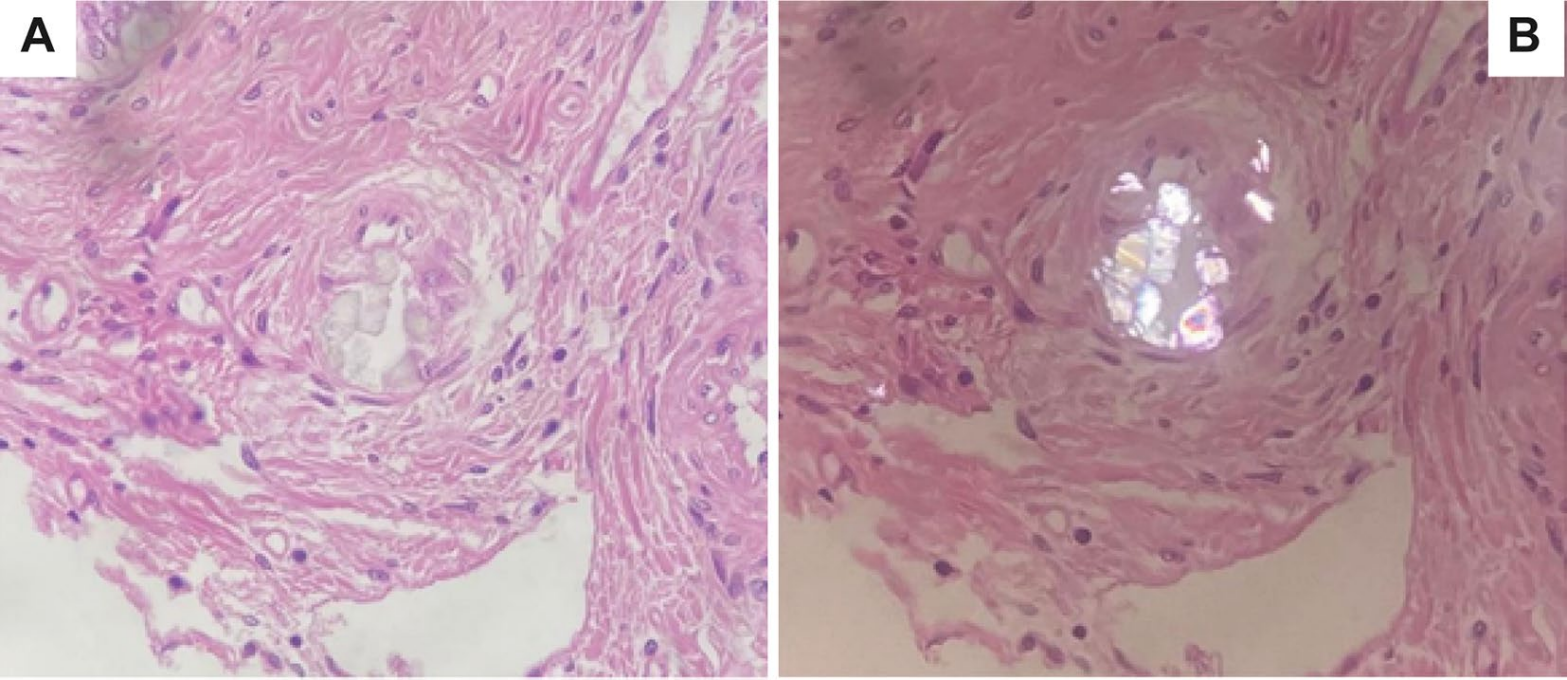
(**A**) Small deposits are visible in the center of the image (patient 17), which are apparently fragmented crystalloid structures with pale grayish color within a portal field (the part above in the picture is still columnar epithelium of a bile duct also included); (HE, 40x). (**B**) The crystalloid structures appear optically polarized birefringent. (Polarization, HE 40x).

The results of all histological examinations are summarized in Table 2. Calcium-oxalate deposition in the liver as typical birefringent crystals was detected by polarization microscopy in three patients. The crystals were located in small arteries, but not within hepatocytes (Figs. 2, 3). Since most CaOx crystals were better preserved after H&E staining, we checked for these crystals in all liver tissue sections with polarized light. Crystal sizes were not uniquely large, as shown in the figures, but were also substantially tinier.

**Table 2:** Results of histological examinations, (+) low specification, + modest specification, + + high specification, CaOx = calcium-oxalate.

Patient N°	1	2	3	4	5	6	7	8	9	10	11	12	13	14	15	16	17	18	**19**
Porto-portal fibrosis					+					+ +									
Nodular fibrosis										+									
Portal fibrosis	+	(+)		(+)		+		+	+			(+)						(+)	
Central fibrosis	(+)		+ +					+	+				+						
Cirrhosis											+								
Unspecific hepatitis (predominantly portal)		+	+		+		+	(+)	+	+ +									
Sinusoidal fiber proliferation		(+)	(+)		+	+	+	+	+	+								(+)	(+)
Myofibroblasts			+																
Portal iron deposits					+ +		+						+						
Sinusoidal and central iron deposits	+				+	(+)	+ +						+ +		+	+	+		+
Extramedullary hematopoiesis		+			+		+												
Unspecific cholangitis							+												
Signs of portal hypertension										+									
CaOx crystals										+	+						(+)

**Figure 4:**
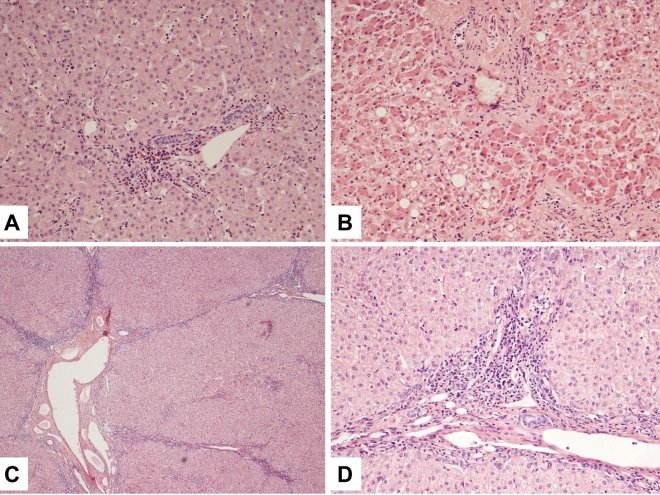
The routine stains showed different types of fibrosis in the biopsy specimen: (**A**) Tiny porto-portal fibrosis together with a lymphocytic infiltration of the portal fields and the adjoining parenchyma in the sense of an interface-hepatitis (patient 5). (**B**) In the autopsy case (patient 11) fibrosis was very advanced towards a cirrhotic remodeling. (**C**, **D**) Porto-portal fibrosis together with a nodular fibrosis (patient 10).

The five routine stains showed different types of fibrosis in the biopsy specimen (Fig. 4). Tiny porto-portal fibrosis together with a leukocytic infiltration of the portal fields and the adjoining parenchyma in the sense of an interface-hepatitis was found in patient 5 (Fig. 4A). In the autopsy case (patient 11) the fibrosis was very advanced towards a cirrhotic remodeling (Fig. 4B). One sample showed a porto-portal fibrosis together with a nodular fibrosis (patient 10, Fig. 4C and D), other cases presented with a limitation of the fibrosis to the portal field (patients 1, 2, 4, 6, 8, 9, 12, 18) and/or to central areas (patients 1, 3, 8, 9 and 13). The fibrosis in the sample of patient 10 was much more distinctive compared to the others. Unspecified hepatitis features were also seen in seven other samples, which were limited to the portal fields (patients 2, 3, 5, 7, 8, 9 and 10).

Sinus fiber proliferations were detectable in nine cases (patients 2, 3, 5–10 and 18). In one sample transformed Ito-cells (myofibroblasts) were observed by α-actin-stain (patient 3).

In addition there were iron deposits as indicators of either hemolysis or red blood cell transfusions. In one case they were seen in larger quantities in the portal fields (patient 5). In 6 other samples there were only some iron deposits located in the sinus and central areas (patients 1, 6 and 15–17, 19). Two further cases showed tiny portal in addition to high-grade sinuous and central iron depositions (patients 7 and 13).

In three patients it was possible to detect megakaryocytes by the special immune-histological stain CD61, which confirmed the presence of extramedullary hematopoiesis (patients 2, 5 and 7).

In patient 11, diagnosis and treatment were so delayed that distinctive systemic oxalosis developed with manifestations especially in kidney, skin, liver, bone marrow and myocardium, as is depicted in Fig. 1 and Table 3. The oxalate crystals in the skin caused vascular ischemic necrosis, which became superinfected in the course of the disease. The damage of the myocardium, caused by CaOx deposition, finally led to heart failure and consecutively death.

**Table 3:** Presence of oxalate crystals in different organs of the autopsy case (patient 11), NAD = (no appreciable disease), + +  = high, +  = modest, (+) = low.

Organ	Histology	Oxalate crystals
Kidney	Progressive glomerulonecrosis, tubule atrophy, interstitial fibrosis	++
Skin (leg)	Superinfected gangrene with attendant venous thrombosis, vasal lumen occluded by crystals	+ +
Liver	Cirrhosis	+
Bone marrow	Local fibrosis, resorbing reaction with foam cells	+
Myocardium	Interstitial fibrosis, hypertrophy of cardiomyocytes	+
Lung	Relapsing pulmonary embolism in small arterys without pulmonary infarction	(+)
Spleen	N. A. D	(+)
Pancreas	N. A. D	(+)
Lymph node	N. A. D	(+)
Artery, elastic type	N. A. D	(+)
Thyroid gland	N. A. D	(+)

## Discussion

Literature reports on systemic oxalosis in patients with PH I give evidence on CaOx deposits and crystal-induced damage in many organs, such as kidneys, bones, vessels, retina, myocardium and even the central nervous system^1–3,21–23^. Although the disease causing enzyme defect of PH I is located in the liver, there is no systematic study searching for structural alteration in the liver of patients with PH I. We were able to retrieve five case reports, at which hepatic CaOx deposition was described to take place in the media of the small, but also bigger arteries, as well as in the connective tissue of the portal areas, but not overtly in the liver parenchyma^24–28^. In one patient micronodular cirrhosis was found, which had, however, not affected the patient^26^. Also, crystal deposition in the gall bladder wall was found in another patient^25^.

Our findings indicate that there is no uniform histological pattern of liver tissue changes in patients with PH I. All specimen showed different types of liver involvement and it is speculative, on whether or not the changes were truly related to PH I or to other, though unclassified reasons.

In accordance to the case reports mentioned above^24–28^, we found CaOx deposition in liver arterioles of only three PH I patients. So, in general, oxalate deposition in the liver occurs, but we would have expected a much more pronounced accumulation in the organ of oxalate overproduction. We, of course, have to consider that the paraffin embedding procedure can lead way to washing out of crystals. However, as seen in the autopsy case with other organs examined after same embedding, e.g. kidney, still a significant amount of crystals is found.

It is remarkable that there is a cirrhotic remodeling in 2 of 3 samples which had CaOx crystal deposition. It is, however, discussable on whether or not there is a causal relationship of these phenomena. Maybe the crystals are a secondary incident, which is caused by the fibrosis, for example by constricting the blood flow in parts of the liver. In result of this, CaOx deposits more easily and forms growing crystals. In turn, the crystals may also constrict the blood flow, damage endothelium and cause a necrosis of the surrounding parenchyma and subsequent fibrosis. Both may later result in a vicious circle.

Because of the correlation of CaOx crystal deposition and liver cirrhosis, it may be interesting to take a special look at the connection between cirrhosis and the course of disease itself. For patient 11, who had hepatic CaOx crystal deposition, the diagnosis of PH I was made only after ESKF had occurred and 27.7 years after the first symptom had appeared. Therefore, this patient did not get an adequate, disease specific therapy and even maintenance HD was not adapted to the needs of PH I: aggressive, more or less daily HD and even additional nightly intermittent peritoneal dialysis^29–31^. In this patient the course of disease was fatal and the tissue damage in the conducting system of the heart, caused by oxalosis, finally led to death (Fig. 1). The other patient with CaOx crystal deposition in the liver (patient 10) was treated by isolated kidney transplantation, without a diagnosis of PH I before transplantation. Before that, she was dialyzed for a long time (108 months). As the PH diagnosis was unknown, the dialysis regimen was also not adequate, as only routine (3 times weekly) hemodialysis was performed. The third patient was on aggressive hemodialysis (6 times 3 h per week) but also for a long period of time (72 months before transplantation). Therefore, the CaOx deposition in liver can be explained either by inadequate dialysis regimens or the long period of dialysis in all cases. It is a known factor also, that the duration of dialysis before transplantation negatively affects the outcome, which, of course, may also lead way to more pronounced oxalate deposition^19^.

Nevertheless, we would have had expected much more crystal deposition in the liver in all patients examined. As oxalate is produced in the liver, the oxalate concentration in hepatic tissue and later in the arteries must be high, which should induce parenchymal deposition, like it does in every other body organ. Also, animal models of hyperoxaluria using^14^C oxalate showed, that oxalate concentration in liver and heart was significantly higher than in plasma^32^. It is well known, that PH I is very heterogenous in its clinical appearances, even within families and with the same genotype^8^. Though, especially in patients with overt systemic oxalosis leading to death, the liver deposits are just minimal. This leaves us speculating, that liver tissue may be protected from crystal attachment or ameliorate the transport of oxalate out of the liver cells promptly into the blood vessels^33^, where the crystals were actually found. In addition, the higher concentration of albumin in the liver and thus increased binding of calcium may reduce the risk of calcium-oxalate crystal development. Also, induction of a cellular response mechanism, thus increased macrophage delivery and destruction of crystals may play a role in reduced crystal deposition. Further research is clearly needed here to better understand this phenomenon, which may be helpful to find new treatment possibilities.

Interestingly, fibrotic remodeling of liver was found in every patient, and fiber proliferation, especially in sinus and central vein, were seen in nearly all patients. In addition, in many samples leukocytic infiltration of the portal fields with predominantly lymphocytes were visible. Both, fiber proliferation and inflammatory infiltrates, but especially fibrosis are signs of true liver damage. However, as we only found hepatic CaOx deposition in three patients and as these deposits were not located in the hepatocytes, but only in the vessel walls, it is questionable on whether the underlying disease (= enzyme defect) was the major culprit? Nevertheless, the histological changes do not appear to be age-related and hence may not be based on other secondary factors.

Overall, to conclude our findings, crystal deposition in the liver of PH I patient only rarely takes place and is restricted to arteries and portal tissue. Nevertheless, liver involvement defined as fibrotic remodeling was more pronounced as previously described. This was, however, clearly not related to the amount of crystal deposition in the liver.

## Data Availability

The patients data are placed in the mentioned registry (IRB votes for that registry available).The histology slides are available on request to corresponding author.
